# Genetic Heterogeneity in Cowpea Genotypes (*Vigna unguiculata* L. Walp) Using DArTseq (GBS)-Derived Single Nucleotide Polymorphisms

**DOI:** 10.3390/genes15060764

**Published:** 2024-06-11

**Authors:** Goitsemang Mahlomola Hendry Dikane, Moosa Mahmood Sedibe

**Affiliations:** Department of Agriculture, Faculty of Health and Environmental Sciences, Central University of Technology, Private Bag x20529, Bloemfontein 9301, South Africa; gdikane@cut.ac.za

**Keywords:** plant breeding, geneticdiversity, genotyping-by-sequencing, homogeneity, heatmap, legumes, marker-assisted breeding, next-generation sequencing, pleiotropy, UPGMA dendrogram

## Abstract

Cowpeas (*Vigna unguiculata* L. Walp) have been credible constituents of nutritious food and forage in human and animal diets since the Neolithic era. The modern technique of Diversity Array Technology (DArTseq) is both cost-effective and rapid in producing thousands of high-throughputs, genotyped, single nucleotide polymorphisms (SNPs) in wide-genomic analyses of genetic diversity. The aim of this study was to assess the heterogeneity in cowpea genotypes using DArTseq-derived SNPs. A total of 92 cowpea genotypes were selected, and their fourteen-day-old leaves were freeze-dried for five days. DNA was extracted using the CTAB protocol, genotyped using DArTseq, and analysed using DArTsoft14. A total of 33,920 DArTseq-derived SNPs were recalled for filtering analysis, with a final total of 16,960 SNPs. The analyses were computed using vcfR, poppr, and ape in R Studio v1.2.5001-3 software. The heatmap revealed that the TVU 9596 (SB26), Orelu (SB72), 90K-284-2 (SB55), RV 403 (SB17), and RV 498 (SB16) genotypes were heterogenous. The mean values for polymorphic information content, observed heterozygosity, expected heterozygosity, major allele frequency, and the inbreeding coefficient were 0.345, 0.386, 0.345, 0.729, and 0.113, respectively. Moreover, they validated the diversity of the evaluated cowpea genotypes, which could be used for potential breeding programmes and management of cowpea germplasm.

## 1. Introduction

Cowpea (*Vigna unguiculata* (L.) Walp.) is a pulse crop grown in the wet and dry agro-ecologies of the tropics, primarily where there is a lack of protein sources for human consumption [1]. It is a source of inexpensive protein, vitamins, and minerals in both rural and urban areas and is a meat protein substitute that is rich in stable carbohydrates [2]. The bulk producer of cowpea grains in sub-Saharan Africa is Nigeria, with approximately 3.6 million metric tons produced in 2021, while consuming more than 3.6 million metric tons [3]. In South Africa, smallholder farmers produce a yield of less than 500 kg per hectare for subsistence demands [2]. The FAO [3] reported a production of approximately 4.87 thousand metric tons in South Africa in 2021.

The productivity of cowpeas (mainly cultivated in semi-arid tropics) may be impeded by irregular rainfall patterns, despite being the most drought-tolerant crop [1]. Heat can severely affect the yield of sensitive varieties because of poor pollen development, which causes flower abortion when temperatures reach 35 °C at night and results in poor seed and pod set [4]. Production is also hampered by soils that lack organic matter and have low phosphorus levels [5].

Cowpeas contain phenolic acids, flavonoids, and tannins, which carry bioactive antioxidant, anti-inflammatory, and anti-carcinogenic compounds that provide health benefits to humans and animals [4]. The use of flavonoids and tannin-rich forages has yielded improved ruminant health and productivity through increased resistance to gastrointestinal nematodes, reduced bloating, and improved nutrition via the presence of high-quality proteins in the small intestines [4]. Cowpea seeds are also said to have cardio-protective potency effects and prevent cardiovascular diseases [4]. Infused seeds treat amenorrhea when taken orally, and, according to the indigenous people of South Africa, crushed roots (when eaten with porridge) are believed to treat painful menstruation, epilepsy, and chest pain [4].

Modern DNA marker technology has been applied to cowpea research programmes for the molecular classification of germplasm, genetic development, and quantitative trait loci (QTL) mapping [6]. Forty-four genotypes from four different subgenera of the genus *Vigna* have been classed using restriction fragment length polymorphism (RFLP) markers [6]. Both wild and cultivated relatives have been further genetically classed for diversity studies using the following markers: random amplified polymorphic DNA (RAPD), inter-simple sequence repeats (ISSRs), amplified fragment length polymorphisms (AFLPs), and simple sequence repeats (SSRs) [6]. Landraces from across the globe and African wild ancestral cowpeas have been collected and genotyped using 1200 single nucleotide polymorphism (SNP) markers to reveal and class gene pools [6]. Recently, SNPs have been discovered with next-generation sequencing (NGS) technologies using genotyping-by-sequencing (GBS) to appropriate genetic diversity, phylogenetic interactions, and population structures [6].

More recently, a high-throughput GBS technology platform known as Diversity Array Technology sequencing (DArTseq) has been used to identify SNPs [7]. It enables high-throughput, whole-genome profiling of crops, without requiring previous DNA sequence information, by using microarray hybridisations that detect present and absent individual fragments in the genomic profile [7]. DArTseq allows for the selection of appropriate complexity-reduction methods for each organism, application, and selected genome assays and has been used for the molecular analyses of the large garlic germplasm bank [8]. Identification of new gene sources for the development of better-performing cowpea breeding lines would require the use of DArTseq molecular screening of existing germplasm and improve breeding lines that are currently available. The main objectives of this study were to assess the genetic diversity of select cowpea genotypes using DArTseq (SNPs) and to identify heterogeneous genotypes and their genomic profiles that could be used for breeding programmes and crop improvements.

## 2. Materials and Methods

### 2.1. Plant Material and Sampling Procedure

Ninety-two genotypes that are commonly used in Nigeria and South Africa for breeding programmes based on their yield potential, seed colour, germination period, and abiotic and biotic stress performance were selected from the germplasm collection at the Agricultural Research Council-Grain Crops (ARC-GC). Four seeds from each cowpea genotype were planted using potting soil and covered by a thin layer of vermiculite to retain moisture in a 200 × 5 cm^2^ pot. The pots were labelled with their genotypes and placed in a greenhouse during the autumn season at the ARC-GC, Potchefstroom, South Africa. Minimum temperatures ranged from 8 °C to 14 °C and the maximum temperatures ranged from 20 °C to 28 °C in a glasshouse. After 13 days of propagation, young trifoliate, fresh, and succulent leaves were excised from four plants and bulked into a single sample from each genotype, totalling to 368 plants. The leaf samples were placed in sterile centrifuge tubes and freeze-dried at −21 °C for five days using a freeze-drying machine. Leaf samples were labelled with their corresponding genotype sample code (Table 1), stored at −81 °C for 10 days, and sent to the Integrated Genotyping Service and Support (IGSS), Bioscience Eastern and Central Africa Hub-International Livestock Research Institute (BecA-ILRI), Nairobi, Kenya, for DNA extraction, DArT sequencing, and SNP analysis.

### 2.2. DNA Extraction

DNA extraction and sequencing were performed using the DArTseq™ protocol (Diversity Arrays Technology Pty Ltd., Canberra, Australia). Approximately 1 g of young leaf tissue from each accession was used for genomic DNA extraction. Genomic DNA was isolated from frozen leaves using a modified cetyltrimethyl ammonium bromide (CTAB)/chloroform/isoamylalcohol method [9]. Frozen leaf tissue was ground and mixed with 2% pre-warmed (60 °C) CTAB isolation buffer containing 1.4 M NaCl, 100 mM Tris pH 8.0, and 20 mM EDTA (Sigma, Saint Louis, MO, USA). The mixture was then transferred to a 2 mL microcentrifuge tube and incubated at 60 °C for one hour. DNA was extracted once with chloroform–isoamyl alcohol (Chl/IAA, 24:1) (Sigma, Saint Louis, MO, USA) and precipitated using two volumes of isopropanol. The obtained pellet was washed with 70% EtOH, dried and dissolved in 100 µL TE buffer with 50 µg/mL RNAse A (Sigma, Saint Louis, MO, USA). The extracted DNA was quantified using 0.8% agarose gel electrophoresis, which was adjusted to 50 ng/μL for DArT and SNP genotyping.

### 2.3. DArTseq Analysis

DNA was processed in digestion/ligation reactions as reported by Kilian [10] by replacing a single PstI-compatible adaptor with two different adaptors corresponding to two different restriction enzymes (REs) as follows: PstI- and SphI-compatible adaptors. The PstI-compatible adapter was designed to incorporate an Illumina flow-cell attachment sequence with staggered sequences of varying length barcode regions, like the sequence reported by Elshire [11]. The reverse adapter contained a flow-cell attachment region with an SphI-compatible overhang sequence. Only mixed fragments (PstI-SphI) were effectively amplified in 30 rounds of PCR using the following reaction conditions: 94 °C for 1 min, then 30 cycles at 94 °C for 20 s, 58 °C for 30 s, 72 °C for 45 s, and 72 °C for 7 min. Following PCR, equimolar amounts of amplification products from each sample of the 96-well microtiter plate were bulked and applied to a c-Bot (Illumina) bridge PCR followed by sequencing on Illumina Hiseq2500 (Illumina, San Diego, CA, USA). The sequencing (single read) was run for 77 cycles.

Sequences generated from every lane were processed using proprietary DArT analytical pipelines (PLs). In the primary pipeline analysis, fragments of poor-quality sequences with reproducibility below 90% and a read depth lower than 3.5 for SNPs or 5 for the presence or absence markers were filtered out. More stringent selection criteria were applied to the barcode region compared with the remainder of the sequences. The assignments of the sequences to specific samples carried within the barcode split step were extremely reliable [12]. No samples were dropped because of low coverage across loci; however, individual sequences were removed if they did not meet the above criteria. Approximately 2.5 million sequences per barcode/sample were identified and used in marker calling. The average browse depth across loci was 9.2 reads per individual per locus for reference alleles and 6.5 for SNP alleles. Finally, identical sequences were collapsed into fastqcoll files. The fastqcoll files were groomed using DArT PL’s proprietary algorithm, which corrects a low-quality base from a singleton tag into a correct base using collapsed tags with multiple members as a template. The groomed fastqcoll files were used in the secondary pipeline for DArT PL’s proprietary SNP and SilicoDArT (presence/absence of restriction fragments in representation; PA markers) calling algorithms using DArTsoft14 (Diversity Arrays Technology Pty Ltd., Canberra, Australia). Two types of DArTseq markers were scored, SilicoDArT markers and SNP markers, which were both scored in a binary fashion for presence/absence (1 and 0, respectively) of the restriction fragment with the marker sequence as the genomic representation of the sample. Both SilicoDArT and SNP markers were aligned to the reference genomes of Vunguiculata_469_v1.0 to identify chromosome positions.

### 2.4. SNP Calling

SNP calling was conducted for all tags from all libraries enclosed within the DArTsoft14 analysis and clustered using DArT PL’s C++ algorithm program at a brink distance of 3. Parsing of the clusters into separate SNP loci was completed by employing a technique called the balance of read counts for the allelic pairs [11]. Additional choice criteria were further added to the algorithm program and supported by an analysis of roughly 1000 controlled cross-populations. Testing for deviations from the Hardy–Weinberg equilibrium of alleles in these populations was conducted to facilitate the selection of technical parameters to effectively discriminate true allelic variants from paralogous sequences. In addition, multiple samples were processed from the DNA to allelic calls as technical replicates and scoring consistency was used as the main selection criteria for high-quality/low-error rate markers. Calling quality was assured by a high average browse depth per locus (the average across all markers was over 30 reads/locus), and DNA was diluted to 50 ng/µL for the GBS analysis [12].

### 2.5. Data Analysis

Statistical R Studio software (v1.2.5001-3) packages, including vcfR, poppr, ape, tidyr, and ggplot, were used to generate the unweighted pair group method with arithmetic mean (UPGMA) dendrogram, principal component analysis, and a hierarchical heatmap. All genetic parameters, including microsatellite loci scoring, were calculated using R Studio (R software version 3.4) for each locus allele frequency (MAF), heterozygosity (observed; Ho, and expected; He), polymorphic information content (PIC), and inbreeding coefficient (F_IS_) [12]. 

## 3. Results

Ninety-two cowpea genotypes were sequenced using SilicoDArT markers (indicating present/absent fragments in DArT genomic representations) and SNP data. A total of 33,920 DArTseq-derived SNPs were recalled for analysis, with only 16,960 reserved for further computed analyses. 

### 3.1. Cluster Dendrogram (UPGMA)

**Figure 1:**
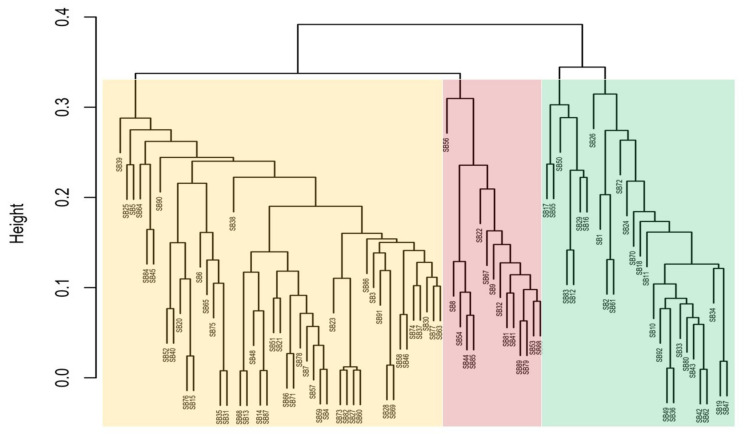
Cluster dendrogram (UPGMA) illustrating the genetic distances of 92 cowpea genotypes. Clusters are illustrated with pale orange, red and green colours.

The UPGMA (unweighted pair method with arithmetic mean) cluster dendrogram results in Figure 1 illustrate three main clusters (indicated by three shaded colours) of genetic distances among 92 genotype SNPs. The first branch of the dendrogram (cluster 1, in yellow) shows the genotypes TVU 13778 (SB39), TVU 12637 (SB25), TVU 3416 (SB5), and Dr Saunders (SB46) to be the most genetically distant within this cluster. Cluster 2 (indicated with a pink colour) shows the genotypes RV 553 (SB56), RV 194 (SB8), and CH-14 (SB22) to be the most genetically distant among genotypes found in the same cluster. Cluster 3 (indicated by a green colour) shows the genotypes RV 403 (SB17), 90K-284-2 (SB55), RV 411 (SB50), TVU 9596 (SB26), TVU 14190 (SB1), and Orelu (SB72) to be the most genetically distant. A general assessment of the 92 branched genotypes indicated that SB39, SB56, and SB26 were the most distantly related genotypes (Figure 1).

### 3.2. Principal Component Analysis

**Figure 2:**
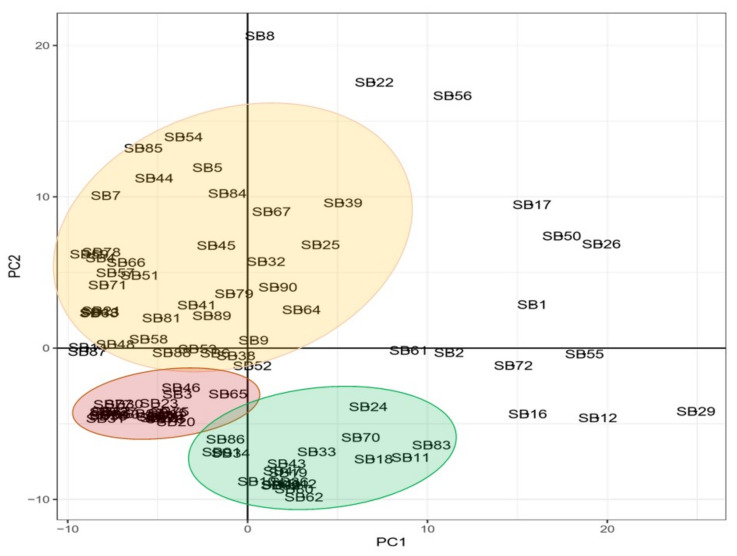
Principal component analysis illustrating the genetic populations of 92 cowpea genotypes. The clustering of genotypes is illustrating by pale orange, red and green colours.

A principal component analysis (PCA) was used to illustrate the grouping and scattering of genotypes based on their genetic relation, as shown in Figure 2. There are three groups of cowpea genotypes situated on the first, third, and fourth quadrants of the two-dimensional PCA, showing genetic similarities by distance. Accessions RV 194 (SB8), CH-14 (SB22), RV 553 (SB56), RV 403 (SB17), RV 411 (SB50), TVU 9596 (SB26), and TVU 14190 (SB1) are genetically distant from the outlined groups, and mainly situated in the second quadrant. Accessions Orelu (SB72), 90K-284-2 (SB55), RV 503 (SB2), RV 442 (SB12), 97K-499-35 (SB29), RV 502 (SB61), and RV 498 (SB16) are genetically distant from the outlined groups and primarily in the 4th quadrant (Figure 2). 

### 3.3. Hierarchical Heatmap

**Figure 3:**
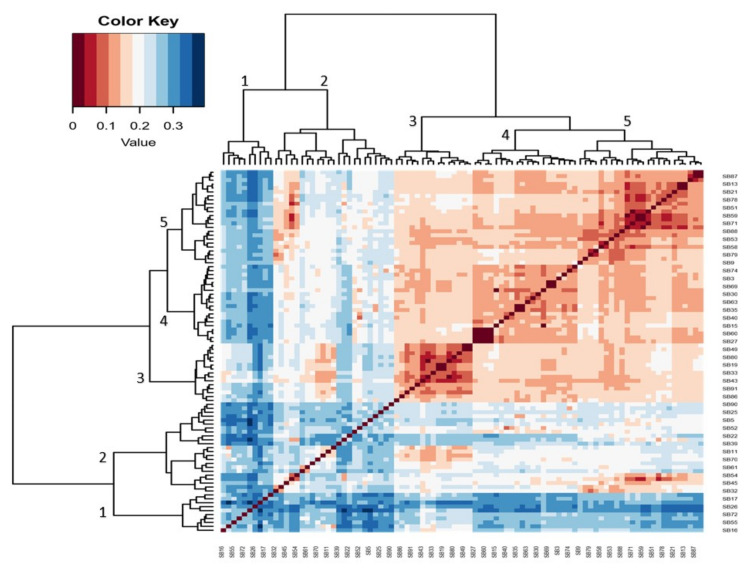
Hierarchical heatmap (correlation matrix) illustrating heterogeneity among the most diverse cowpea genotypes.

The hierarchical heatmap shown in Figure 3 further classed five main clusters of 46 genotypes that were more genetically distant among the 92 genotypes illustrated in Figure 1. Values below 0.2 show less genetic distance and those above 0.2 show more genetic distance among the genotypes. The genotypes with moderate genetic distances shown in cluster 2 are CH-14 (SB22), TVU 13778 (SB39), TVU 3416 (SB5), RV 500 (SB90), RV 502 (SB61), and TVU 12637 (SB25) (Figure 3). The genotypes with more genetic distance shown in cluster 1 are TVU 9596 (SB26), Orelu (SB72), 90K-284-2 (SB55), RV 403 (SB17), and RV 498 (SB16).

### 3.4. Heterogeneity Analysis

Ninety-two cowpea genotypes were sequenced to attain single-nucleotide polymorphic base pairs that were equi-frequent at a specific genomic locus on a chromosome. Gene diversity among cowpea genotypes was assessed (Table 2) using major allele frequencies (MAFs), which yielded an average of 0.729 (range 0.719–0.739). Observed heterozygosity (Ho) averaged 0.386 with a range of 0.358–0.413. Expected heterozygosity (He) averaged 0.345 (range 0.345–0.346), and polymorphic information content (PIC) averaged 0.345 with a range of 0.333–0.354. The inbreeding coefficient (FIS) averaged −0.113 with a range of −0.167 to −0.052, as indicated in the table below (Table 2).

## 4. Discussion

The DArTseq-SNP data were analysed by computing a UPGMA dendrogram, which uncovered three major clusters with clear genetic diversity among the selected genotypes. The analysis also revealed significant genetic distance relations, both individually and in populations, that can be used in germplasm selection for breeding objectives and germplasm maintenance (Figure 1). The PCA confirmed some of the distinct genotypes indicated in the previous analysis (Figure 2). The heatmap analysis illustrated half of the genotypes that possessed the greatest genetic distance to narrow down the selection of divergent parental genotypes (Figure 3). Genotypes were grouped into clusters based on their genetic distances. The most divergent parental genotypes were TVU 9596 (SB26), Orelu (SB72), 90K-284-2 (SB55), RV 403 (SB17), and RV 498 (SB16). These findings agree with Malik [13] and indicate that PCAs can be successfully used to classify and measure patterns of genetic diversity in germplasm. Moreover, PCA has been effective in assessing genetic diversity for agro-morphological traits in chickpea genotypes [14].

A marker possessing two or more alleles with 99% frequency is qualitatively referred to as polymorphic [15]. The extent of polymorphism is quantitatively measured by heterozygosity and the polymorphism information content (PIC) value [15]. Polymorphisms can be classed into three categories as applied in simple-sequence repeats (SSRs) studies including PIC > 0.5 (highly informative), 0.5 < PIC > 0.25 (moderately informative), and PIC < 0.25 (slightly informative) [16]. This study demonstrated the genetic diversity of genomic DArTseq-SNPs distributed among eleven chromosomal loci of ninety-two cowpea genotypes, with a mean PIC value of 0.345 and a range of 0.333–0.354 (moderately informative polymorphism). This observation could have been caused by low mutations and the bi-allelic nature of SNPs [17]. In population genetic analyses, major and minor allele frequencies are determined at a particular locus in a population to indicate genetic diversity [12]. Our study focused on the most common alleles for given DArTseq-SNPs in a population. The major allele frequency had a significant mean value of 0.729 with a range of 0.719–0.739. The result inferences are based on frequencies that are MAF ≥ 0.5% [18]. Inferences with values close to 1 predict a better distribution of allele frequencies at a given locus in the observed population [12]. The inbreeding coefficient (F_IS_) was also used as a measure of the non-random association of alleles in individuals in a population. This study recorded a F_IS_ mean value of −0.113 with a range of −0.167 to −0.052. Negative F_IS_ values indicate the presence of heterozygotes within individuals in a population [19]. 

Heterozygosity is the most important parameter used to estimate genetic diversity in a population. Gene diversity, also known as expected heterozygosity (He), outlines expected genotypes that are heterozygous under Hardy–Weinberg equilibrium [16]. The recorded mean value of He was 0.345 (range 0.358–0.345) and the observed heterozygosity (Ho) mean value was 0.386 with a range of 0.358–0.413. Both mean observed and expected heterozygosity showed moderate genetic variation, as supported by a mean negative inbreeding degree. The mean He was relatively equal to the PIC, and both were less than Ho. This could be a result of the evenness of allele frequencies, indicating individuals that could have identical heterozygote genotypes [17]. 

Genetic variability in plant material is the basic information needed for breeders to improve crops by adopting an appropriate method of selection [20]. This study highlights the DArTseq platform as an approach for genome complexity reduction. The platform provides whole-genome sequencing, which covers adequate genomic information per locus, improved genotype calling, and the ability to sequence more samples for a lower cost [21]. The system can be applied without available reference genomes using double-digest restriction fragment sequencing, which appropriates evolutionary genetic studies in the bio-system [21]. DArTseq is a cost-effective genotyping platform used for maintaining, profiling, ascertaining, and managing available biodiversity in germplasm banks. DArTseq sequences can be used to develop DArTseq markers and other molecular markers such as SNPs or SSRs, which can be used in other germplasm banks [8]. This information can be further used in association-of-trait-of-interest and marker-assisted breeding. 

The DArTseq GBS platform was used by the CIMMYT on their seeds of discovery project, which profiled more than 40,000 wheat gene bank genotypes, as reported by Li [22]. The DArTseq platform produces high throughput molecular markers that help to discriminate the gene pools of cowpeas by detecting substantial genetic variation among genotypes [12]. DArTseq SNPs are important in the selection of diverse parental genotypes in crosses because of their capability to discriminate gene pools. Breeding programmes can narrow the genetic variation in crops. Thus, cowpea germplasm that is introduced and moved to a new environment could increase genetic variation. While arable land for farming is limited because of globalisation, this propels the genotypic advancements of crops with improved genetic variation to guide breeding programmes [6].

## 5. Conclusions

DArTseq provided high throughput SNP markers that illustrate the heterogeneity in the selected cowpea genotypes from the ARC-GC cowpea germplasm collection. DArTseq SNPs provided whole genome information with a good distribution of major allele frequencies on observed genotypes, which is beneficial when selecting parents with novel genes from the current collection with known agronomic traits. This technology has also supplied negative inbreeding coefficients among individual genotypes in a population within a short time. The SNPs could be further used to construct high-resolution novel gene mapping experiments and to identify genes responsible for traits of interest that can be used in genomic-wide association studies. 

Genotypes TVU 9596 (SB26), Orelu (SB72), 90K-284-2 (SB55), RV 403 (SB17), and RV 498 (SB16) could be used as divergent parents in breeding programmes. Furthermore, the overall genetic heterogeneity in the selected genotypes indicated moderately informative polymorphisms. These cowpea genotypes are recommended for use in the development of cowpea markers, and they have pre-recorded, agro-morphological attributes that can qualify them for the improvement and characterisation of other genotypes that are not genetically screened in the ARC-GC germplasm collection. 

## Figures and Tables

**Table 1:** List of cowpea genotypes used for DArTseq genotyping and genetic diversity analysis.

Genotype Sample Code	Genotype Name	Origin	Genotype Sample Code	Genotype Name	Origin	Genotype Sample Code	Genotype Name	Origin
SB1	TVU 14190	Nigeria	SB32	RV 351	South Africa	SB63	ARC 039	South Africa
SB2	RV 503	South Africa	SB33	98K-503-1	Nigeria	SB64	ARC 022	South Africa
SB3	TVU 2095	Nigeria	SB34	RV 126	South Africa	SB65	ARC 029	South Africa
SB4	BECHUANA WHITE	South Africa	SB35	TVU 11424	Nigeria	SB66	AGRINAWA	South Africa
SB5	TVU 3416	Nigeria	SB36	RV 447	South Africa	SB67	RV 343	South Africa
SB6	PAN 311	South Africa	SB37	RV 558	South Africa	SB68	ARC 012	South Africa
SB7	RV 204	South Africa	SB38	TVU 11986	Nigeria	SB69	ARC 020	South Africa
SB8	RV 194	South Africa	SB39	TVU 13778	Nigeria	SB70	RV 327	South Africa
SB9	RV 438	South Africa	SB40	98D-1399	Nigeria	SB71	ARC 040	South Africa
SB10	95K-589-2	Nigeria	SB41	RV 341	South Africa	SB72	ORELU	Nigeria
SB11	RV 416	South Africa	SB42	RV 440	South Africa	SB73	RV 315	South Africa
SB12	RV 442	South Africa	SB43	OLOYIN	Nigeria	SB74	RV 417	South Africa
SB13	RV 207	South Africa	SB44	RV 202	South Africa	SB75	ARC 037	South Africa
SB14	ARC 045	South Africa	SB45	ENCORE 349	South Africa	SB76	RV 439	South Africa
SB15	ARC 024	South Africa	SB46	DR SAUNDERS	South Africa	SB77	ARC 009	South Africa
SB16	RV 498	South Africa	SB47	RV 568	South Africa	SB78	ARC 004	South Africa
SB17	RV 403	South Africa	SB48	RV 554	South Africa	SB79	RV 352	South Africa
SB18	98K-476-8	Nigeria	SB49	RV 446	South Africa	SB80	RV 457	South Africa
SB19	IT93K-1294	Nigeria	SB50	RV 411	South Africa	SB81	RV 361	South Africa
SB20	RV 329	South Africa	SB51	ARC 006	South Africa	SB82	ARC 013	South Africa
SB 21	RV 157	South Africa	SB52	83S-911	Nigeria	SB83	ARC 026	South Africa
SB22	CH 14	South Africa	SB53	RV 342	South Africa	SB84	TVU 13953	Nigeria
SB23	TVU 12746	South Africa	SB54	ARC 008	South Africa	SB85	TVU 13998	Nigeria
SB24	TVU 9443	South Africa	SB55	90K-284-2	Nigeria	SB86	IT00K-1263	Nigeria
SB25	TVU 12637	South Africa	SB56	RV 553	South Africa	SB87	RV 555	South Africa
SB26	TVU 9596	South Africa	SB57	RV 213	South Africa	SB88	RV 344	South Africa
SB27	ARC 014	South Africa	SB58	GLENDA	South Africa	SB89	CH 47	South Africa
SB28	86D-1010	Nigeria	SB59	ARC 005	South Africa	SB90	RV 500	South Africa
SB29	97K-499-35	Nigeria	SB60	RV 320	South Africa	SB91	98K-506-1	Nigeria
SB30	RV 321	South Africa	SB61	RV 502	South Africa	SB92	97K-207-15	Nigeria
SB31	99K-494-6	Nigeria	SB62	ARC 025	South Africa			

**Table 2:** Heterogeneity assessment of 92 cowpea genotypes using DArTseq-derived SNPs.

Chromosome No.	No. of SNPs	MAF	He	Ho	PIC	FIS
1	518	0.7283853	0.3462423	0.3856441	0.3462469	−0.10820243
2	499	0.7300559	0.3462424	0.3804404	0.3428320	−0.10128670
3	1016	0.7357895	0.3462425	0.3783386	0.3433586	−0.09397598
4	729	0.7189496	0.3462426	0.3922707	0.3538314	−0.10568182
5	654	0.7252793	0.3462427	0.4037193	0.3439191	−0.16628110
6	680	0.7239614	0.3462428	0.4054283	0.3518747	−0.14416821
7	882	0.7327958	0.3462429	0.3667573	0.3469722	−0.05202273
8	630	0.7234917	0.3462430	0.4132967	0.3522159	−0.16792216
9	563	0.7329730	0.3462431	0.3895356	0.3357911	−0.15095750
10	687	0.7318918	0.3462432	0.3770733	0.3453611	−0.08789979
11	639	0.7393492	0.3462433	0.3577612	0.3331006	−0.06902656
Mean	681.5455	0.7293566	0.3450427	0.3863878	0.3450458	−0.1134023
SE	46.0903	0.001815176	0.001949046	0.005066684	0.001948986	0.01172541

Note: MAF = major allele frequency, Ho = observed heterozygosity, He = expected heterozygosity (genetic diversity), PIC = polymorphic information content, FIS = inbreeding coefficient, No. of SNPs = number of single nucleotide polymorphisms, SE = standard error.

## Data Availability

The data are contained within this article.
